# Examining Long-Term Effects of Human Papillomavirus Vaccine Recommendation Messages: A 4-Month Follow-Up Survey of a Randomized Controlled Study in Japan

**DOI:** 10.3390/healthcare8040549

**Published:** 2020-12-10

**Authors:** Tsuyoshi Okuhara, Hirono Ishikawa, Haruka Ueno, Hiroko Okada, Takahiro Kiuchi

**Affiliations:** 1 Department of Health Communication, School of Public Health, The University of Tokyo, Tokyo 113-8655, Japan; uenoh-tky@umin.ac.jp (H.U.); okadahiroko-tky@umin.org (H.O.); tak-kiuchi@umin.ac.jp (T.K.); 2 School of Public Health, Teikyo University School of Medicine, 2-11-1 Kaga, Itabashi-ku, Tokyo 173-8605, Japan; hirono-tky@umin.ac.jp

**Keywords:** human papillomavirus (HPV) vaccination, anti-vaccination movement, narrative, persuasion, long-term effect, health communication

## Abstract

We previously conducted a randomized controlled study to examine persuasive messages recommending HPV vaccination to mothers with daughters in Japan. That study showed that the three types of intervention message used (statistical information only, a patient’s narrative in addition to statistical information, and a mother’s narrative in addition to statistical information) all significantly improved mothers’ intention to have their daughter(s) receive the HPV vaccine, in comparison with mothers who received no messaging. The present study is a follow-up survey to assess the long-term effect of the intervention. Four months after the initial study, in January 2018, participants in the previous study were contacted and queried about their current intention to have their daughter(s) receive the HPV vaccine. Statistical analysis was conducted using the paired *t*-test and analysis of variance. A total of 978 mothers participated in the current survey. Vaccination intention 4 months after intervention had decreased to a level that did not differ significantly from the level prior to intervention in all three intervention conditions. The amount of change in vaccination intention 4 months after intervention did not differ significantly among the three intervention groups (*p* = 0.871). A single exposure to messaging was insufficient to produce a persistent intervention effect.

## 1. Introduction

The human papillomavirus (HPV) vaccines have contributed to reduce the public health burden of cervical cancer [1,2,3]. However, widespread uptake of the vaccine is necessary for this advance. The situation is critical in Japan. Proactive recommendation of HPV vaccination was suspended by the Ministry of Health, Labour and Welfare in June 2013 in Japan after negative campaigns by mass media about severe adverse reactions allegedly caused by HPV vaccination [4]. Since then, the HPV vaccination rate among age-eligible girls has been stagnating, with only 0.3 percent of girls being vaccinated [5]. Individuals have fears concerning adverse reactions to HPV vaccination in Japan and other countries [6,7,8,9,10], although studies have demonstrated the safety and the efficacy of HPV vaccines [2,3,11]. To sway this biased anti-HPV vaccination sentiment in this critical situation, influential communication tactics are needed to encourage the public to make less biased decisions.

Educational interventions to increase HPV vaccination acceptance have been conducted using media such as information fact sheets, leaflets, slide presentation, and educational videos [12,13]. In those media, statistical and narrative evidences offer the advantage of being easy to use when health professionals create HPV vaccination recommendation messages. Several studies in the context of vaccine communication show that narrative messages about experiences of disease increase the audience’s risk perception of developing the disease, vaccination intention, and behaviors to prevent the disease to a greater degree than do didactic messages [14,15,16,17]. In the current Japanese situation where side reactions to HPV vaccines are concerned, presenting statistical data on risks and benefits of HPV vaccines will be essential to support audiences’ balanced decision making regarding vaccination, and presenting only narrative messages may be neither sufficient nor desirable. Therefore, HPV vaccination recommendation messages in Japan for the time being may be two ways: using only statistical evidence, or adding narrative evidence to statistical evidence. However, few studies have compared message persuasiveness between a combination of narrative and statistical evidence and statistical evidence alone [18]. The only study that examined it―presenting messages of non-health-related topic to undergraduate students―showed that a combination of narrative and statistical evidence was more persuasive than statistical evidence alone [19]. No study has examined whether a combination of narrative and statistical evidence was more persuasive than statistical evidence alone in HPV vaccination communication in non-student samples.

Therefore, we previously conducted a randomized controlled study to examine the types of persuasive messages to use when recommending HPV vaccination to mothers with daughters in September 2017 in Japan [20]. We compared message persuasiveness according to four conditions: messages including statistical information only; statistical messages plus narrative messages of a patient who experienced cervical cancer; statistical messages plus narrative messages of a mother whose daughter experienced cervical cancer; and a control condition [20]. Our previous study showed that messages containing statistics information only, as well as a narrative (of a patient or a mother) in addition to statistical messages, significantly increased participants’ attitude and intention to have their daughter(s) receive the HPV vaccine, compared with prior to the intervention and with participants who received no messaging (*p* < 0.001) [20]. This result indicated that exposure to messaging about efficacy and safety of HPV vaccine may increase vaccination intention of mothers with daughters directly after exposure to messaging, whether this includes only statistical messages, or narratives of experiences with cervical cancer in addition to statistical messages. Additionally, our previous study showed that the intention for the statistical messages plus mother’s narrative was the highest among the intervention groups and was significantly higher than in the statistics-only group (*p* = 0.040) [20]. This indicated that the narrative messages of a mother whose daughter experienced cervical cancer may be persuasive for audiences who are mothers with daughters.

Our previous study only assessed participants’ vaccination intention directly after exposure to messaging. The long-term effects of messages should be investigated because HPV vaccination expects multiple injections given over a series of weeks: In Japan, the second dose is given 1 month after and the third dose is given 6 months after the first dose. One previous study examined the impact of an individually tailored intervention on knowledge, risk perception, intention to be vaccinated, and uptake regarding HPV vaccine, among unvaccinated female university students over 3 months following the intervention [21]. However, no studies have examined the long-term effects of intervention among parents of adolescent daughters. Therefore, in the present study, we contacted participants in our previous study 4 months after the initial investigation and reassessed their intention to have their daughter(s) receive the HPV vaccine. The present study aimed to examine whether the effects of intervention persisted 4 months after the intervention, and whether the degree of persistence of the intervention effect depends on the type of message. We set the following research questions.

Research question 1: Does vaccination intention 4 months after intervention differ significantly from the intention before intervention?

Research question 2: Does the amount of change in vaccination intention 4 months after intervention differ significantly among the three types of message?

## 2. Materials and Methods

### 2.1. Summary of the Previous Study

#### 2.1.1. Participants and Procedures

Detailed information was reported in our previous study [20]. Briefly, in that study, participants were recruited from among individuals registered in a survey company database in Japan. Eligibility criteria were mothers with a daughter(s) aged 12–16 years who had never received HPV vaccination. A total of 1432 mothers completed our previous web-based survey in September 2017. Participants were randomly assigned to a group that received statistical messages only (*n* = 394), a group that received a message that included statistical information plus a patient’s narrative (*n* = 408), a group that received statistical information plus a message including a mother’s narrative (*n* = 411), or a control group that received no message (*n* = 219), using an algorithm included in the survey computer program. All participants were asked sociodemographic information, history of cancer and sexually transmitted diseases, and whether they knew about the media coverage regarding adverse reactions to the HPV vaccine and suspension of the proactive recommendation for HPV vaccination by the Japanese government. Regarding the outcome, intention to have one’s daughter(s) receive the HPV vaccine was assessed before and directly after intervention.

#### 2.1.2. Intervention Materials

Regarding the intervention materials, the statistical information conveyed morbidity and mortality of cervical cancer and efficacy and safety of HPV vaccine. The content was identical in the intervention materials presented to the three groups. For the narrative content, the narrator recounted their experience of being diagnosed with cervical cancer, giving up having children, developing from complications, fearing cancer recurrence, and recommending HPV vaccination. The narrative contents were identical for the intervention materials presented to the groups receiving a patient’s and mother’s narratives, except the subject of the narrative differed (i.e., “I” in the patient’s narrative and “my daughter” in the mother’s narrative). The statistical message and mother’s narrative used in our previous study are provided in Appendix A, which was translated into English for this report.

### 2.2. Methods of the Present Study

#### 2.2.1. Participants and Procedures

Four months after the initial study, we conducted a follow-up survey. All of 1432 participants in our previous study were contacted by e-mail and recruited to participate in the follow-up survey in January 2018. Those who consented to participate completed this follow-up survey online. A total of 978 mothers participated in this follow-up survey; the response rate was 68%. Participants were asked about their intention to have their daughter(s) receive the HPV vaccine. In other words, participants reported their vaccination intention before and directly after the intervention in our previous study, as well as 4 months after the intervention in the present study.

#### 2.2.2. Measures

As in our past study, participants’ intention to vaccinate was measured according to their responses to the following three questions: (1) “How likely are you to have your daughter(s) receive the HPV vaccine sometime soon?”; (2) “If you were faced with the decision of whether to have your daughter(s) receive the HPV vaccine today, how likely is it that you would choose to have her receive the vaccine?”; and (3) “How likely is it that you would have your daughter(s) receive the HPV vaccine in the future?”. The following responses were given 1 to 6 points, respectively: “very unlikely”, “unlikely”, “somewhat unlikely”, “somewhat likely”, “likely”, and “very likely”. A mean score was used in the analysis. Higher scores indicated greater intention to vaccinate. This measure was adapted from a previous publication [22].

#### 2.2.3. Sample Size Calculation

Although the present study is a follow-up survey of our initial study [20], we conducted sample size calculations for considering whether the number of participants in the present study produce sufficient statistical power. Based on our initial study [20], we estimated a small effect size (Cohen’s d = 0.20) for research question 1 and a small effect size (f = 0.10) for research question 2 in the current study. We conducted power analyses at an alpha error rate of 0.05 (two-tailed) and a beta error rate of 0.20. The power analyses indicated that 199 participants were required in a group for research question 1 and 323 participants were required in each of the intervention groups for research question 2.

#### 2.2.4. Statistical Analysis

The Cronbach’s α value was used to determine internal reliability of the measures. Descriptive statistics were used to summarize participants’ sociodemographic information and history, using percentage for categorical variables and mean ± standard deviation (SD) for continuous variables. To assess if there was any difference between those who did and did not participate in the 4-month follow-up survey, sociodemographic information and history, baseline vaccination intention, and vaccination intention directly after intervention were compared between followed-up participants and dropouts, using the two sample *t* test and the Chi-square test. Then, participants’ sociodemographic information and history in the present study were compared among groups using the chi-square test, Fisher’s exact test, and analysis of variance (ANOVA). Participants’ vaccination intention was compared between before and 4 months after intervention, using the paired *t*-test. When no variable differed significantly among groups, ANOVA was conducted with changes in the intention to vaccinate before and 4 months after intervention as the dependent variable and the group assignment as the independent variable. Tukey’s test was conducted on the significant main effects, where appropriate. The Games-Howell post-hoc test was performed when the assumption of homogeneity of variances was not satisfied. When there was a variable that significantly differed among groups, analysis of covariance (ANCOVA) was conducted with changes in the intention to vaccinate before and 4 months after intervention as the dependent variable, the group assignment as the independent variable, and the variable that significantly differed among groups as the covariate. Bonferroni correction was applied for the post hoc test to compare adjusted means of intentions. A *p*-value of <0.05 was set as significant in all statistical tests. All statistical analyses were performed using IBM SPSS Statistics for Windows, Version 21.0 (IBM Corp., Armonk, NY, USA).

#### 2.2.5. Ethical Consideration

The protocol was approved by the ethical review committee of the Graduate School of Medicine, The University of Tokyo (No. 11624). All participants gave their written informed consent in accordance with the Declaration of Helsinki.

## 3. Results

### 3.1. Participant Characteristics

There was no significant difference except for mean age between followed-up participants and dropouts (Table A1). A total 258 participants were included in the group that received statistical messages only, 269 were in the group that received a patient’s narrative plus statistical messages, and 284 were in the group that received a mother’s narrative plus statistical messages, and 167 in the control group. Those number of participants were about 70 more than the required sample size for the research question 1, and about 50 less than the required sample size for the research question 2. Table 1 shows the participants’ characteristics. Participants’ age ranged from 31 to 61 years (mean 44 years, SD 4.5). A total 52% of participants’ daughters were 12–14 years old and the remainder were 15–16 years old. About 90% of participants had not been advised by health professionals that their daughter(s) should receive the HPV vaccine and knew about the media coverage regarding adverse reactions to the HPV vaccine and suspension of the proactive recommendation for HPV vaccination by the Japanese government. About 90% of participants did not have a history of cervical cancer or sexually transmitted diseases. Participants’ characteristics were not significantly different among groups.

### 3.2. Intervention Effect

**Figure 1:**
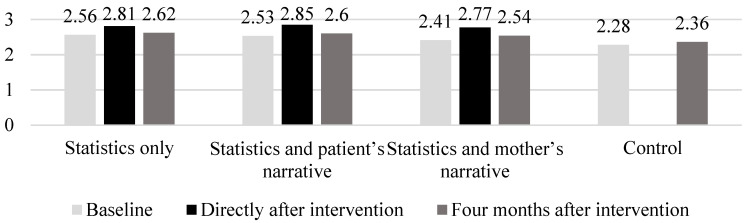
Mean of vaccination intention at baseline, directly after, and four months after intervention across groups.

The internal consistency of questions regarding the intention to have one’s daughter(s) receive the HPV vaccine was excellent (Cronbach’s α = 0.947). The mean scores for participants’ intention to have their daughter(s) receive the HPV vaccine before, directly after, and 4 months after intervention in the three intervention groups were 2.51 (SD 0.95), 2.82 (SD 0.99), and 2.59 (SD 0.97), respectively. The mean of intention in the control group was 2.28 (SD 0.91) in our previous study, and 2.36 (SD 0.92) in the present study. Figure 1 shows the mean vaccination intention at baseline, directly after, and 4 months after intervention across the groups. The mean vaccination intention decreased from directly after intervention to 4 months after intervention: 2.82 (SD 1.00) to 2.62 (SD 1.00) in the statistics only group; 2.84 (SD 1.01) to 2.60 (SD 0.97) in the statistics plus patient’s narrative group; and 2.78 (SD 0.96) to 2.54 (SD 0.95) in the statistics plus mother’s narrative group. Table 2 shows changes in the intention to vaccinate before intervention and 4 months after intervention in the three intervention groups. The paired *t*-test revealed that vaccination intention 4 months after intervention did not differ significantly from the intention before intervention in the intervention groups (*p* = 0.256 in the statistics-only group, *p* = 0.054 in the statistics plus patient’s narrative group, *p* = 0.096 in the statistics plus mother’s narrative group). Also, in the control group, vaccination intention 4 months after intervention did not differ significantly from the intention before intervention (*p* = 0.147). ANOVA revealed no main effect of group assignment on the amount of change in vaccination intention 4 months after intervention (*F*(2.808) 0.138, *p* = 0.871); this indicated that the amount of change in vaccination intention 4 months after intervention did not significantly differ among the three intervention groups.

## 4. Discussion

We examined whether the effect of an intervention recommending HPV vaccination for mothers with daughters had persisted 4 months after the intervention and whether the degree of persistence of the intervention effect depended on the type of message: a statistical message only, a patient’s narrative in addition to statistical messages, or a mother’s narrative in addition to statistical messages.

The results of the present study showed that vaccination intention in all groups slightly increased, although those changes were not significant. The effect of intervention had not persisted at 4 months after intervention, and the degree of persistence of the intervention effect 4 months after intervention did not differ among the three message types. More precisely, vaccination intention 4 months after intervention decreased to a level that did not significantly differ from the level before intervention under all three intervention conditions. Thus, the answer to our research question 1 was “no”.

The amount of change in vaccination intention 4 months after intervention did not differ significantly among the three message types. Thus, the answer to our research question 2 was also “no”. Our previous study showed that vaccination intention increased significantly directly after reading intervention materials and that statistical messages plus a mother’s narrative increased vaccination intention the most [20]. However, the present findings revealed that those effects wore off during the 4 months following intervention. Our results are consistent with those of a previous study showing that risk perception, intention to be vaccinated, and HPV vaccine uptake did not differ significantly between baseline and 3 months following intervention among female university students in the United States [21].

HPV vaccination expects multiple injections given over a series of weeks. In Japan, the second dose is given 1 month after the first dose, and the third dose is given 6 months after the first dose. Studies indicate that there are a number of anti-HPV vaccination websites on the internet [23] and that seeing anti-vaccination messages online can negatively affect the audience’s attitude toward vaccination, even 5 months after exposure to these messages [24]. Additionally, the current COVID-19 pandemic may exacerbate any existing problems with equity in vaccination [25]. During the COVID-19 pandemic, HPV vaccination programs are delayed in countries with clusters of cases and/or community transmission of COVID-19 to minimize the spread of infection [26]. People whose initiation of HPV vaccination has been delayed or whose vaccination schedule has been interrupted due to the COVID-19 pandemic may experience a decrease in vaccination intention during that period. Considering this, persistently increased vaccination intention after an intervention to promote HPV vaccines is important, to effectively conduct vaccination.

To maintain the intervention effect, intervention messages should be easy to remember so that they can be recalled over time by the message recipients [27]. Studies indicate that narratives are the preferred mental structure for storing and retrieving information [28,29], and narratives are better recalled than didactic content [30,31]. Events and characters in narrative messages are linked to each other through personal, causal, temporal, and spatial associations, which facilitate storage and retrieval of more complex information because the recipient need only remember a single story rather than miscellaneous information [32]. Studies indicate that health materials with narrative messages enhance recall [28,29] and are associated with lower decision conflict than messages without narratives [33,34]. However, the advantage of narratives for memory and recall did not seem to be present in this study, considering that the intervention effect of narratives in addition to statistical messages diminished over time to the same degree as the effect of statistical messages only. The reason may be that the narratives used in the present study were created for research purposes and thus may have been too short and uninteresting to be remembered and recalled over time by participants. It may be useful to examine the persuasiveness of a longer and more vivid narrative of an experience of cervical cancer in future studies, as the narrative of a patient or a mother is more likely to be retained and recalled and will consequently influence decision making regarding vaccination over time. Additionally, the present study did not examine how much participants were exposed to the anti-HPV vaccination messages in the media and they believed those negative stories. Anti-HPV vaccination messages may have been influential enough to reduce vaccination intentions, which were improved by the intervention, over the course of 4 months. Future studies should also investigate participants’ exposure to anti-vaccination messages and their perception during the follow-up period.

A review of studies about media exposure indicate that the degree of exposure to health messaging among message recipients is positively associated with the likelihood of their engagement in health behaviors [35,36,37]. It is considered that repeated exposure to health messages enhances recognition and recall of messages among recipients and encourages them to undertake certain health behaviors [38]. To improve persistence of the intervention effect of messages recommending HPV vaccination, it may be important for health institutes to frequently disseminate messages such that target populations are repeatedly exposed to those messages. However, the mean vaccination intention directly after intervention was less than 3 (i.e., somewhat unlikely) in our previous study [9]. Studies reported that knowing the negative news about the HPV vaccine was the main reason why mothers did not get their daughters vaccinated in Japan [39,40]. Frequent messaging alone may be insufficient to increase vaccine uptake. Messaging coupled with other interventions, such as facilitating access to the vaccine and providing educational programs, may help to improve vaccine uptake.

Additional studies indicate that educational programs on topics such as anti-stigma toward mental illness and self-management of chronic illness have improved participant’s knowledge, attitude, and practices over time [41,42,43]. A few studies have examined the effect of educational programs in the context of HPV vaccination after the suspension of governmental recommendation. One study in Japan reported that although educational intervention promoted fathers’ positive attitudes towards HPV vaccination, the intervention did not increase their intention to get their daughters vaccinated [44]. Because acceptance of the HPV vaccine is associated with mother’s perceptions of risk and benefits of vaccination [40], future studies will be expected to examine the effect of educational intervention to mothers. Another study provided educational lectures on HPV vaccine to health science teachers in a Japanese university and found that educational lectures improve their vaccine confidence and recommendation rates for the HPV vaccine to their female students [45]. A mother’s consultation with a doctor is related to the mother’s decision to get her daughter vaccinated in Japan [39]. Educational interventions to health science teachers and doctors, who have influence to girls and mothers, may be more effective to improve and maintain acceptance of HPV vaccines for a long period of time than only disseminating written messages.

Several limitations should be considered in this study. First, as mentioned earlier, it should be noted that factors other than intervention, such as exposure to media coverage, may have influenced vaccination intention among some participants during the 4 months after intervention. Second, when the persuasive intent is obvious, narrative persuasion is hindered because some audiences may resist if they feel they are being manipulated [46]. This constraint may be related to the negative results of the present study and should be noted, in addition to the brief content of the materials, as discussed above. Third, we did not examine the duration of the intervention effect, i.e., how many weeks did the intervention effect persist? Investigating this issue is necessary so as to determine the appropriate frequency of message exposure to sustain increased vaccination intention. Fourth, we assessed vaccination intention rather than vaccine uptake. However, behavioral intention is generally measured in public health studies because it predicts an actual behavior [47]. Fifth, whether some participants had their daughter(s) receive HPV vaccines after intervention is unknown. However, the influence of this on the study results is considered to be small because the HPV vaccination rate is only a few percent in Japan. Finally, the response rate in the present study was 57%; the intention of the 43% of participants who did not respond to the follow-up survey are unknown. The respondents in the present study could be self-selected in the follow-up survey. Selection bias may have influenced the study results.

## 5. Conclusions

Our previous study showed that HPV vaccination intention increased significantly directly after intervention using statistical messages only, a patient’s narrative in addition to statistical messages, and a mother’s narrative in addition to statistical messages. However, the present study showed that the effect of intervention wore off during the 4 months following intervention and that the degree of persistence of the intervention effect 4 months after intervention did not differ among the three message types. It is important that increased vaccination intention is maintained because HPV vaccination expects multiple injections given over a series of weeks. To maintain the intention of HPV vaccination, a single message exposure may be insufficient.

## Figures and Tables

**Table 1:** Participant sociodemographic information and history.

Sociodemographic Information and History	Total (*n* = 978)	Statistics Only (*n* = 258)	Statistics and Patient’s Narrative (*n* = 269)	Statistics and Mother’s Narrative (*n* = 284)	Control (*n* = 167)	*p*
Age, mean year (SD)	43.9 (4.5)	43.9 (4.5)	43.5 (4.3)	44.2 (4.9)	43.8 (4.3)	0.429 ^b^
Age of daughters, %						
12–14 years old	51.5	57.0	49.8	49.1	49.7	0.234 ^c^
15–16 years old	48.5	43.0	50.2	50.9	50.3	
Highest education, %						
Less than high school	3.1	2.7	2.6	4.6	1.8	0.583 ^d^
High school graduate	29.7	30.6	29.7	27.7	31.7	
Some college	42.4	39.5	40.1	44.2	47.3	
College graduate	23.8	25.6	26.4	22.8	18.6	
Graduate school	1.0	1.6	1.1	0.7	0.6	
Household income, %						
Less than 2 million yen ^a^	7.9	7.0	7.4	8.8	8.4	0.917 ^c^
2–6 million yen ^a^	35.5	32.9	36.4	37.2	35.3	
More than 6 million yen ^a^	43.4	47.7	42.4	40.0	44.3	
Unknown	13.2	12.4	13.8	14.0	12.0	
Advised by health professionals to have their daughter(s) receive HPV vaccines, %						
Yes	6.1	8.9	5.2	6.0	3.6	0.124 ^c^
No	93.9	91.1	94.8	94.0	96.4	
Knew about media coverage of adverse reactions to HPV vaccines, %						
Yes	90.1	87.2	90.7	89.5	94.6	0.091 ^c^
No	9.9	12.8	9.3	10.5	5.4	
Knew about suspension of the proactive recommendation for HPV vaccination by the government, %						
Yes	86.2	83.7	87.4	85.6	89.2	0.391 ^c^
No	13.8	16.3	12.6	14.4	10.8	
History of cervical cancer including familiar persons, %						
Yes	8.3	7.4	7.8	9.5	8.4	0.629 ^d^
No	91.2	92.2	91.1	90.5	91.0	
No answer	0.5	0.4	1.1	0	0.6	
History of cancer other than cervical cancer including familiar persons, %						
Yes	18.2	16.3	21.9	16.1	18.6	0.595 ^d^
No	81.5	83.3	77.7	83.5	81.4	
No answer	0.3	0.4	0.4	0.4	0	
History of sexually transmitted disease including familiar persons, %						
Yes	8.1	8.1	10.4	7.0	6.0	0.263 ^d^
No	90.8	91.9	88.1	91.2	92.8	
No answer	1.1	0	1.5	1.8	1.2	

HPV, human papillomavirus; SD, standard deviation. ^a^ One US dollar is roughly equivalent to 100 yen. ^b^ ANOVA. ^c^ Chi-square test. ^d^ Fisher’s exact test.

**Table 2:** Changes in intention of vaccination before and four months after intervention across groups.

**Intention of Vaccination**	**Statistics Only (*n* = 258)**			**Statistics and Patient’s Narrative (*n* = 269)**			**Statistics and Mother’s Narrative (*n* = 284)**			***p*^b^**
	**Before**	**Four Months After**	***p*^a^**	**Before**	**Four Months After**	***p*^a^**	**Before**	**Four Months After**	***p*^a^**	
Intention of vaccination, mean (SD)	2.58 (0.94)	2.62 (1.00)	<0.256	2.53 (0.94)	2.60 (0.97)	<0.054	2.47 (0.95)	2.54 (0.95)	<0.096	
Changes in intention before and four months after intervention, mean (SD)	0.047 (0.663)			0.077 (0.651)			0.068 (0.682)			0.871

SD = standard deviation. ^a^ *p*-values were assessed using the paired *t*-test. ^b^ A *p*-value was assessed using ANOVA.

## Abbreviations

SD	standard deviation
HPV	human papillomavirus
ANOVA	analysis of variance
ANCOVA	analysis of covariance

## Appendix A

The intervention material used in the condition of a mother’s narrative messages in addition to statistical messages.

**About cervical cancer**

Cervical cancer is a disease caused by human papillomavirus (HPV) infection. HPV is transmitted by sexual intercourse and persistent HPV infection can progress to cervical cancer. Approximately 10,000 people are diagnosed with and about 3,000 people die of cervical cancer annually in Japan. Mortality due to cervical cancer has increased, and in recent years patients in their 20–30s are mainly affected.

**About HPV vaccines**

Girls can prevent HPV infection and cervical cancer by being vaccinated with HPV vaccines. Recommended age targets for vaccination are girls in the 6th grade of elementary school to those in the 1st grade of high school.

**Efficacy of HPV vaccines**

Efficacy data on HPV vaccines are now very strong.

■ Many countries (65) have included HPV vaccines in their national immunization programs.
■ Several of these countries (e.g., Australia, USA, Denmark, and Scotland) have reported that the incidence rate of precancerous lesions of the uterine cervix has decreased by approximately 50% since institution of widespread HPV immunization programs.

**Safety of HPV vaccines**

HPV vaccine safety has been confirmed in domestic and foreign surveys.

■ In a domestic survey in Japan, reports of alleged adverse events were made in 2584 cases out of a total of 8.9 million HPV vaccine doses (0.03% of total doses). Of those reporting adverse events, approximately 90% have had complete recovery (186 persons are still receiving medical care related to adverse events). In short, 2 people out of 100,000 administered doses (0.002%) have reported long-term health effects.
■ Safety data using a large-scale re-examination survey conducted by the European Medicine Agency (EMA) and in France revealed no difference in the occurrence rates of severe adverse reactions caused by HPV vaccines between vaccinated and unvaccinated cohorts. The same result was found in a study conducted by Nagoya City in Japan.

**Table A1:** Comparison between followed-up participants and dropouts.

Sociodemographic Information, History and Baseline Vaccination Intention	Followed-Up (*n* = 978)	Dropouts (*n* = 454)	*p*
Age, mean year (SD)	43.9 (4.5)	44.5 (5.0)	0.035 ^b^
Age of daughters, %			
12–14 years old	51.3	46.0	0.062 ^c^
15–16 years old	48.7	54.0	
Highest education, %			
Less than high school	3.2	3.1	0.775 ^c^
High school graduate	29.6	31.7	
Some college	42.4	43.2	
College graduate	23.8	21.4	
Graduate school	1.0	0.7	
Household income, %			
Less than 2 million yen ^a^	7.8	7.5	0.619 ^c^
2–6 million yen ^a^	35.7	35.9	
More than 6 million yen ^a^	43.4	41.0	
Unknown	13.2	15.6	
Advised by health professionals to have their daughter(s) receive HPV vaccines, %			
Yes	6.1	7.7	0.265 ^c^
No	93.9	92.3	
Knew about media coverage of adverse reactions to HPV vaccines, %			
Yes	90.1	90.1	0.997 ^c^
No	9.9	9.9	
Knew about suspension of the proactive recommendation for HPV vaccination by the government, %			
Yes	86.2	85.2	0.629 ^c^
No	13.8	14.8	
History of cervical cancer including familiar persons, %			
Yes	8.3	10.1	0.386 ^c^
No	91.2	89.6	
No answer	0.5	0.2	
History of cancer other than cervical cancer including familiar persons, %			
Yes	18.1	18.9	0.854 ^c^
No	81.6	80.6	
No answer	0.3	0.4	
History of sexually transmitted disease including familiar persons, %			
Yes	7.9	9.5	0.434 ^c^
No	91.0	89.9	
No answer	1.1	0.7	
Baseline vaccination intention, mean (SD) ^d^	2.52 (0.94)	2.45 (0.93)	0.217 ^b^
Vaccination intention directly after intervention, mean (SD)	2.73 (1.00)	2.73 (0.99)	0.902 ^b^

HPV, human papillomavirus; SD, standard deviation. ^a^ One US dollar is roughly equivalent to 100 yen. ^b^ Two sample *t* test. ^c^ Chi-square test. ^d^ Number of followed-up participants and dropouts was 814 and 399, respectively, because baseline vaccination intention was not assessed in the control group.
